# Comparative analysis of the trauma care profile before and during the COVID-19 pandemic: a cross-sectional study in a tertiary university hospital

**DOI:** 10.1590/0100-6991e-20233449-en

**Published:** 2023-02-17

**Authors:** JULIA SANTA CRUZ GOBETTI, MARIAM BLEIBEL ZRAIK, CAMILA BECKMANN AFORNALI, CAIO HENRIQUE MARCHETTE GOVEIA, CARLOS ROBERTO NAUFEL, GUILHERME ANDRADE COELHO, SUELEN GEISEMARA BARCELAR NUNES, EDUARDO BOLICENHA SIMM

**Affiliations:** 1 - Faculdade Evangélica Mackenzie do Paraná, Curso de Medicina - Curitiba - PR - Brasil; 2 - Hospital Universitário Evangélico Mackenzie, Pronto-socorro - Curitiba - PR - Brasil

**Keywords:** Emergencies, COVID-19, Pandemics, Wounds and Injuries, Trauma Centers, Emergências, COVID-19, Pandemias, Ferimentos e Lesões, Centros de Traumatologia

## Abstract

**Objectives:**

to evaluate the profile of emergency care of trauma patients at Hospital Universitário Evangélico Mackenzie (HUEM) during the period of restrictive measures due to COVID-19 (03/13/2021 to 04/05/2021), and compare to the same period at the beginning of the pandemic, in 2020, and before the pandemic, in 2019.

**Methods:**

quantitative and descriptive observational cross-sectional study. The final sample of 8,338 was analyzed in terms of date, gender, age and service responsible for providing care; the traumas were analyzed according to the etiology and conduct of the treatment and outcome.

**Results:**

there was a percentage increase in non-traumatic emergency care during the pandemic, and the medical clinic held a third of admissions in 2021. There was a reduction in trauma care, since in 2019 traumas were responsible for 44.9% of admissions and by 23.5% in 2021. There was a significant difference in the proportion between the attendance of men and women, and the percentage of men victims of trauma was higher than in the pre-pandemic periods. There was a reduction in absolute numbers, with statistical significance, in traffic accidents, falls from the same level, burns, general blunt trauma and sports and leisure trauma. The proportion of conservative treatments with hospital discharge reduced. There was a significant difference in the number of deaths, decreasing in 2020 but increasing in 2021.

**Conclusion:**

there was a reduction in trauma care during the pandemic, but the profile remained the adult male victim of a traffic accident. More severe traumas were admitted, resulting in an increase in surgical treatment, hospitalizations and deaths.

## RESUMO

**Objetivo:**

avaliar o perfil de atendimento emergencial dos pacientes vítimas de trauma do Hospital Universitário Evangélico Mackenzie (HUEM) durante o período de medidas restritivas devido à COVID-19 (13/03/2021 a 05/04/2021) e comparar ao mesmo período no início da pandemia, em 2020, e antes da pandemia, em 2019.

**Métodos:**

estudo transversal observacional quantitativo e descritivo. A amostra final de 8.338 foi analisada quanto a data, sexo, idade e serviço responsável pelo atendimento; os traumas foram analisados conforme a etiologia e a conduta do tratamento e desfecho.

**Resultados:**

houve aumento percentual no atendimento a urgências não traumáticas durante a pandemia, e a clínica médica deteve um terço das admissões em 2021. Ocorreu redução nos atendimentos por trauma, visto que em 2019 os traumas foram responsáveis por 44,9% das admissões e por 23,5% em 2021. Houve diferença significativa na proporção entre os atendimentos de homens e mulheres, sendo que o percentual de homens vítimas de traumas foi maior do que os períodos pré pandêmicos. Observou-se redução em números absolutos, com significância estatística, nos eventos de trânsito, queda de mesmo nível, queimaduras, traumas contusos gerais e traumas esportivos e de lazer. A proporção de tratamentos conservadores com alta hospitalar reduziu. Houve diferença significativa na quantidade de óbitos, reduzindo em 2020, mas aumentando em 2021.

**Conclusão:**

houve redução do atendimento no trauma durante a pandemia, mas o perfil permaneceu sendo o homem adulto vítima de eventos de trânsito. Traumas de maior gravidade foram admitidos, resultando no aumento de tratamento cirúrgico, internamentos e óbitos

## INTRODUCTION

Coronavirus disease 2019 (COVID-19), an acute respiratory infection caused by SARS-CoV-2, was discovered in December 2019 in China and was declared a pandemic on March 11, 2020, by the World Health Organization^1^ ^-^ ^3^. According to the Oswaldo Cruz Foundation (Fiocruz), Brazil faced the biggest health and hospital collapse in its history, with overcrowding of the health system. Due to the need to reduce the circulation of the virus, Curitiba, in the State of Paraná, adopted restrictive measures for circulation, in accordance with Decree No. 565, of 03/13/2021. Thus, it declared a public health emergency on 03/16/2020, extending until 04/05/20214.

Social restrictions seem to have repercussions on several areas of health, including trauma. International studies sought to understand the epidemiology of traumatic injuries in times of social distancing rules and identified a change in the care profile. A reduction of 31-62% in the total number of admissions was observed in periods of social restriction compared with similar periods before the pandemic. In addition, there was an increase in trauma due to high level falls (70% compared with the previous year), representing the predominant type of trauma found during the lockdown period^5^ ^-^ ^16^.

Despite the reduction in the number of patients admitted due to trauma to emergency departments, an upward trend was observed in the proportion of severe traumas^8^ ^,^ ^11^ ^-^ ^13^. Rajput et al. observed that, in England, there were more high-risk incidents and a higher mortality rate compared with the same period in 2019 (7.4%-6.2%)^12^. In addition, there was an increase in patients who required emergency surgery, while the number of patients who did not need intervention reduced in proportion during lockdown. The hypothesis would be that the apprehension of exposure to SARS-CoV-2 postponed care, resulting in therapeutic delay and more aggressive interventions^12^ ^-^ ^14^.

The profile of trauma victims in Brazil before the pandemic was of men of reproductive age victims of traffic events^17^ ^-^ ^19^. The main trauma mechanisms were due to car accidents (50.3%), falls (22.5%), and interpersonal violence (21.2%)^17^. However, in view of the scarcity of national studies, the profile during the pandemic is not well understood. Ribeiro-Junior et al. sought to understand the changes in trauma and violence during the initial stages of the pandemic in São Paulo. They noted a reduction in injuries from traffic events and a drop in the number of injuries from firearms and stab weapons. Data related to sexual violence and interpersonal violence also declined. However, these numbers may be relative, due to the tendency of underreporting of cases of domestic violence, since the search for help in these cases is usually impaired^20^.

The objective of this study was to evaluate the emergency care profile of trauma victims at the Hospital Universitário Evangélico Mackenzie - a tertiary health service in Curitiba - Paraná, during the period of more restrictive measures in the city - 03/13/2021 to 05/05 04/2021 - and to compare it to the same period at the beginning of the pandemic, in 2020, and, also before the pandemic, in 2019.

## METHODS

This is a quantitative, descriptive, observational, cross-sectional study, with non-probabilistic sampling, which obtained information through electronic medical records from the Hospital Universitário Evangélico Mackenzie (HUEM), a Type 3 trauma center in Curitiba, Paraná, Brazil.

In view of the different people movement patterns during the COVID-19 pandemic, information was collected from all patients who were treated in the HUEM emergency sector in three similar periods of consecutive years: 03/13 to 05/04, 2019 (before the pandemic), 03/13 to 05/04, 2020 (in the beginning of the pandemic), and 03/13 to 05/04, 2021 (during the pandemic’s most restrictive measures). 

We collected data on date, sex, age, and service responsible for providing care. The sample was 11,436, and of these, 3,098 were excluded because they were pediatric appointments.

Regarding sex, the sample was divided between females and males.

As for the service responsible for the care, the sample was divided into 8 groups: internal medicine, gynecology and obstetrics, non-trauma general surgery, non-trauma ophthalmology, non-trauma orthopedics, trauma general surgery, trauma ophthalmology, and trauma orthopedics. To analyze traumatic emergencies, the last three groups were joined and configured the trauma group. This group was further analyzed, firstly regarding the main etiology: traffic events, fall from the same level (FSL), fall from another level (FAL), burn, blunt trauma, penetrating trauma, and others; and regarding the circumstance of occurrence: sports and leisure trauma, violence, and unspecified. Traffic events and interpersonal violence were classified, where applicable, the former into run over and events by motor vehicles, bicycles, or motorcycles, and the latter into self-harm, assault, domestic violence, and sexual violence. We also evaluated the trauma group regarding conduct and treatment outcome: conservative discharge, conservative hospitalization, surgery, or death.

If the patient had more than one etiology and/or was approached by more than one service, the main etiology and/or the service that carried out the main procedures was considered. Separate visits by the same patient were considered separate episodes.

We analyzed the collected data using descriptive statistics with tables. The research was developed respecting the privacy of the participants. Personal data that could identify the patient (name, address, education level, and documentation) were not collected. The study was approved by the Ethics in Research Committee of the institution (Opinion No. 4803371).

To carry out the statistical analysis, we tabulated the information from the medical records in an Excel spreadsheet for the descriptive analysis and performed the chi-square and Fischer tests using the SPSS Statistics v.28.0 software.

## RESULTS

We analyzed data from 8,338 emergency medical records during the three periods, 2,757 in 2019, 2,449 in 2020, and 3,132 in 2021. Non-traumatic emergency room visits increased in percentage during the pandemic, internal medicine accounting for almost a third of all admissions in 2021. On the other hand, there was a progressive reduction in trauma visits, which were responsible for 44.9% of admissions in 2019 (n=1237), 43.1% in the initial period of the pandemic (n=1055), and, though displaying the highest absolute number of attendances, represented only 23.5% in 2021 (n=737) (Tables 1 and 2).

**Table 1:** Attendances by specialty according to years.

Service	2019		2020		2021		
	n	%	n	%	n	%
IM	557	20.2%	511	20.9%	985	31.4%
GO	257	9.3%	243	9.9%	592	18.9%
Non-trauma GS	465	16.9%	319	13.0%	473	15.1%
Non-trauma Ophthalm	128	4.6%	216	8.8%	196	6.2%
Non-trauma Ortho	113	4.1%	106	4.3%	149	4.7%
Trauma GS	767	27.8%	700	28.6%	411	13.1%
Trauma Ophthalmic	71	2.6%	63	2.6%	62	2.0%
Trauma Ortho	399	14.5%	291	11.9%	264	8.4%
TOTAL	2757	100%	2449	100%	3132	100%

IM: internal medicine; GO: gynecology and obstetrics; GS: general surgery; Ophthalm: ophthalmology; Ortho: orthopedics. Source: authors.

**Table 2:** Comparison of periods according to age.

Year	Age (years)					
	n	Average	Median	Minimum	Maximum	Standard deviation
2019	1237	41,7	38	18	96	17,9
2020	1055	41,8	38	18	95	18,2
2021	737	40,8	37	18	90	16,8

One-way ANOVA, p<0.05. Source: authors.

For the next analyses, we considered only cases of traumatic emergencies.

Regarding age, the median was 38 years old in the first two periods and 37 in 2021 (p=0.412) (Table 2).

In the analysis of the proportion between sexes, men constituted the largest portion of the sample during the years 2019, 2020, and 2021 - 61%, 66.4%, and 63.6%, respectively. When comparing the initial stage of the pandemic with the same period in the previous year, there was a significant difference in the proportion between men and women attended (p=0.027), and the percentage of male trauma victims was even higher.

Regarding trauma etiologies and circumstances, we observed a tendency towards reduction in absolute numbers, being significant (p<0.001) for traffic events, FSL, burns, general blunt traumas and sports and leisure traumas. Although decreasing in number, traffic events, general blunt trauma, and FSL were the most frequent etiologies in all periods, traffic events remaining in first place (Table 3).

**Tabela 3:** Categorical variables according to years.

Variable	Classification	Year						
		2019		2020		2021		
		n	%	n	%	n	%	p
Trauma etiology	Traffic events	309	24.9%	275	26.1%	167	22.6%	<0.001
	FSL	255	20.6%	201	19.0%	132	17.9%	<0.001
	FAL	86	6.9%	84	7.9%	79	10.7%	0.855
	Burn	185	14.9%	150	14.2%	93	12.6%	<0.001
	General blunt trauma	274	22.2%	198	18.7%	162	22.0%	<0.001
	Penetrating trauma	74	5.9%	98	9.3%	77	10.4%	0.127
	Others	54	4.4%	49	4.7%	27	3.7%	0.008
Trauma circumstance	Sports and leisure Trauma	26	2.1%	13	1.2%	6	0.8%	0.001
	Violence	92	7.4%	98	9.3%	87	11.8%	0.719
	Not specified	1119	90.5%	944	89.5%	644	87.4%	<0.001
Type of traffic event	Run over	34	11.2%	24	8.8%	13	7.9%	0.009
	Automobile	69	22.7%	40	14.7%	25	15.2%	<0.001
	Bicycle	31	10.2%	29	10.7%	31	18.9%	0.957
	Motorcycle	170	55.9%	179	65.8%	95	57.9%	<0.001
Type of violence	Self-harm	6	6.5%	7	7.2%	8	9.2%	0.866
	Assault	79	85.9%	80	82.5%	69	79.3%	0.6146
	Domestic violence	4	4.3%	9	9.3%	7	8.0%	0.386
	Sexual violence	3	3.3%	1	1.0%	3	3.4%	0.564

Chi-square test, p<0.05. Source: authors.

As for the types of traffic events, only those involving bicycles did not display a significant difference. Traffic events involving automobiles decreased in the two pandemic years (n=69 vs. n=40 vs. n=25). On the other hand, motorcycle events increased in 2020 and decreased in 2021. Finally, the proportion of injuries involving bicycles increased 8.7% from 2019 to 2021. Sports and leisure injuries decreased from 2.1% to 0.8 % of total admissions in the years 2019 and 2021, respectively (Table 3).

Table 4 distinguishes the variables regarding the sex of the victim over the years. There was a significant increase in penetrating trauma among women in all periods (n=7 vs. n=14 vs. n=26) (p=0.002). With regard to self-harm, before the pandemic, most cases were female, representing 66.7%. In 2020, 85.7% were men, though with no statistically significant difference (Table 4).

**Table 4:** Categorical variables according to years and sex.

			Sex (%)					
Variable	Classification	Total (n)	2019		2020		2021	
			Female	Male	Female	Male	Female	Male
Trauma etiology	Traffic Event	309	27.2	72.8	20.7	79.3	24	76
	FSL	255	58.4	41.6	55.2	44.8	57.6	42.4
	FAL	86	41.9	58.1	36.9	63.1	38	62
	Burn	185	53.3	46.5	45.3	54.7	34.4	65.6
	General blunt trauma	274	33.9	66.1	28.3	71.7	34	66
	Penetrating trauma	74	9.5	90.5	14.3	85.7	33.8	66.2
	Others	54	25.9	74.1	34.7	65.3	40.7	59.3
Trauma circumstance	Sports and leisure trauma	26	7.7	92.3	7.7	92.3	33.3	66.7
	Violence	92	25	75	21.4	78.6	32.2	67.8
	Not specified	119	40.6	59.4	35.1	64.9	37.7	62.3
Traffic event type	Run over	34	47.1	52.9	41.7	58.3	23.1	76.9
	Automobile	69	47.8	52.2	42.5	57.5	44	56
	Bicycle	31	32.3	67.7	6.9	93.1	29	71
	Motorcycle	170	14.1	85.9	15.6	84.4	17.9	82.1
Type of violence	Self-harm	6	66.7	33.3	14.3	85.7	50	50
	Assault	79	15.2	84.8	12.5	87.5	23.2	76.8
	Domestic violence	4	100	0	100	0	71.4	28.6
	Sexual violence	3	100	0	100	0	100	0
	Surgical	151	39.7	60.3	33.6	66.4	27.5	72.5
Conduct and outcome	Conservative - discharge	970	38.5	61.5	35	65	39.1	60.9
	Conservative - admission	39	30.5	69.2	21.7	78.3	38.1	61.9
	Death	9	55.6	44.4	25	75	20	80

Fisher’s exact test or Chi-square test, p<0.05. Source: authors.

There was a significant difference in the number of deaths (p=0.038), reducing in the first moment of the pandemic, but increasing significantly in 2021. The proportion of conservative treatments with hospital discharge decreased, while hospitalizations and surgeries increased (Table 5).

**Table 5:** Treatment and outcome.

		2019		2020		2021		p
Conduct and outcome	Surgical	151	12.9%	146	14.0%	153	21.4%	0.973
	Conservative - discharge	970	83.0%	824	79.0%	506	70.7%	<0.001
	Conservative - admission	39	3.3%	69	6.6%	42	5.9%	0.004
	Death	9	0.8%	4	0.4%	15	2.1%	0.038

Chi-square test, p<0.05. Source: Copyright.

## DISCUSSION

The present study sought to analyze the profile of trauma care at a tertiary university hospital during the pandemic.

Regarding the total number of admissions, the year 2021 had the highest one, though displaying the lowest number of traumas. When considering that, in 2021, social distancing measures were more restrictive, the high number of emergency room visits (n=3,132) was surprising. However, we observed a significant increase in internal medicine cases, which may be related to the advance of the pandemic and greater circulation of the virus. The number of traumas decreased by 40.4% between 2019 and the period of greatest social restriction, corroborating Fahy et al., who described a reduction of 40.0%^7^. This considerable reduction may be related to the reallocation of beds to accommodate patients with COVID-19.

Most of the analyzed studies found an increase in the median age of trauma victims^7^ ^,^ ^9^ ^,^ ^11^ ^,^ ^13^ ^,^ ^14^, which does not corroborate this study, in which there was no significant difference in the median between years.

Regarding the proportion of attendance between men and women, the data were conflicting. As the pandemic wore on, even more men were victims of trauma, while Fahy et al. found an increase in the proportion of women^7^. Other studies did not show changes in this proportion^6^ ^,^ ^14^ ^,^ ^21^.

A study carried out in Curitiba, in 2012, found that traffic events represented the most prevalent trauma etiology^17^. This prevalence did not change during the pandemic, although the absolute number has decreased, which can be explained by the lower use of cars due to circulation restrictions, since a good part of the workers and educational institutions were paralyzed or in remote activities. It is noteworthy that several international studies did not show traffic events as the main cause of attendance at trauma centers^5^ ^-^ ^7^ ^,^ ^9^ ^,^ ^11^ ^,^ ^13^ ^-^ ^15^ ^,^ ^21^.

With regard to motorcycle events, there was an increase of 9.9% in the first moment of the pandemic, possibly due to the greater demand for home deliveries. In addition, there was a percentage increase in bicycle events, from 10.2% in 2019 to 18.9% in 2021. A possible explanation would be the use of bicycles, both as a mode of home delivery and for leisure and sport. According to data from the Brazilian Association of the Bicycle Sector, purchases of this means of transport increased by 50.0% during the pandemic^22^.

As found by Dolci et al., Hampton et al., and Van Aert et al., sports and leisure trauma suffered a significant decrease (p<0.001). In this study, the reduction was 76.9%^6^.

As for penetrating trauma, the proportion between sexes changed drastically between the first and last analyzed periods, revealing an increase of 24.3% in women. Despite this, we found no association between this finding and cases of domestic violence, which did not suffer a significant difference (p=0.311).

A Brazilian study conducted in the early stages of the pandemic, using official violence data, found a reduction in interpersonal and sexual violence in São Paulo^20^. We found a decrease only in assaults, which went from 79 cases in 2019 to 62 in 2021. On the other hand, self-injury increased mainly among males, by 200.0% in 2020. Our study corroborates the finding of Nia et al., who showed a 300.0% increase in the suicide rate among men during the pandemic^11^. The increase in this form of violence appears to be associated with higher rates of social isolation, which indicate a greater risk for the occurrence of self-harm, since they are linked to feelings of loneliness, resulting in anxiety, depression, and suicidal thoughts^23^. In addition, cases of unemployment and economic losses were also part of the context of the pandemic and, associated with all the uncertainty of a completely new moment, may be related to the increase in self-harm. Even with this plausible explanation, it is possible that this significant percentage increase was due to the low number of consultations for this trauma, resulting in a greater sensitivity to variations.

As found in studies carried out in Austria and the United States, there was a trend towards more severe trauma, as surgical treatments, hospitalizations, and deaths increased^8^ ^,^ ^11^. Possibly, with the reallocation of beds, the hospital admitted the most serious traumas as a priority, while the milder cases were referred to other services. With the pandemic, centers specializing in elective surgeries were adapted to receive trauma victims, due to the lower availability of beds in hospitals and the cancellation of elective surgeries. Motta et al. demonstrated the conversion of a Brazilian institute specializing in high-complexity, elective orthopedic procedures into a trauma care unit during the pandemic period^24^.

## CONCLUSION

Through the present study, we can conclude that the pandemic resulted in a reduction in trauma care, but the predominant profile remained the adult male victim of traffic events. In addition, more severe traumas were admitted, resulting in an increase in surgical treatments, hospitalizations, and deaths.

Therefore, despite the need to reallocate beds and concentrate forces in the fight against the COVID-19 pandemic, trauma remains a public health problem, which requires the maintenance of an adequate hospital structure.
